# Causal determinants of gout in 614,000 adults: A two-sample Mendelian randomization study of dietary, lifestyle, and metabolic traits

**DOI:** 10.1097/MD.0000000000050619

**Published:** 2026-09-11

**Authors:** Mohammad A. Jareebi

**Affiliations:** a Department of Family and Community Medicine, College of Medicine, Jazan University, Jazan, Saudi Arabia.

**Keywords:** body mass index, causal inference, FinnGen, gout, hyperuricemia, Mendelian randomization, triglycerides, UK Biobank

## Abstract

Gout affects over 55 million people worldwide, with prevalence projected to rise by 70% by 2050. Although observational studies have implicated several dietary, lifestyle, and metabolic risk factors, causal relationships remain uncertain because of confounding and reverse causation. Univariable and multivariable two-sample Mendelian randomization (MR) analyses were performed using genome-wide association data from 2 European cohorts: UK Biobank (6543 self-reported gout cases and 456,390 controls) and FinnGen (3576 cases and 147,221 controls). Genetic instruments for 12 exposures, including dried fruit, salad, and cheese intake; smoking initiation; physical activity; body mass index (BMI); and lipid traits, were derived from established genome-wide association study datasets. Inverse-variance weighted regression with multiplicative random effects was the primary analysis, complemented by weighted median, weighted mode, MR-Egger, MR-Pleiotropy RESidual Sum and Outlier, and 3 predefined multivariable models. Benjamini-Hochberg correction was applied across all 24 tests. BMI showed the strongest and most consistent causal effect on gout (FinnGen: odds ratio [OR] = 1.97, 95% confidence interval [CI] = 1.51–2.57; UK Biobank: OR = 1.006, 95% CI = 1.003–1.009; both *P* < .001) and remained significant in 5 of 6 multivariable models. Triglycerides increased risk in both cohorts (FinnGen: OR = 1.34, 95% CI = 1.08–1.66; UK Biobank: OR = 1.008, 95% CI = 1.004–1.012). These associations survived Benjamini-Hochberg correction, as did high-density lipoprotein cholesterol in UK Biobank (OR = 0.997, 95% CI = 0.994–0.999). Dried fruit intake was inversely associated with gout in FinnGen (OR = 0.34, 95% CI = 0.13–0.92, *P* = .034) but not in UK Biobank, and did not survive correction for multiple testing. No other exposure reached significance in either cohort. This study provides genetic evidence that BMI is the dominant modifiable causal determinant of gout, with triglycerides contributing independently. Dietary associations were weaker and did not withstand correction for multiple testing, and should be regarded as hypothesis-generating. These findings support prioritizing weight management and metabolic health in gout prevention.

## 1. Introduction

Gout represents a rapidly expanding global health challenge, affecting an estimated 55.8 million individuals worldwide as of 2020, with prevalence projected to rise by over 70% by 2050.^[1]^ This complex metabolic disorder and common form of inflammatory arthritis affects 1% to 4% of the global population, with a disproportionate impact on males at a 3:1 ratio, though postmenopausal women face a 2- to 3-fold higher risk than their premenopausal counterparts.^[2,3]^ The condition’s burden intensifies with age, reaching an incidence of 11.8% by age 75, making it a significant public health concern across diverse populations.^[4,5]^

The pathophysiology of gout centers on purine metabolism dysregulation, wherein uric acid accumulates in the blood, leading to hyperuricemia. When uric acid concentrations exceed physiological thresholds, crystallization occurs in joints and surrounding tissues, triggering acute inflammatory episodes characterized by intense pain, erythema, and swelling, most commonly affecting the first metatarsophalangeal joint.^[6]^ While dietary purine content has traditionally guided risk assessment, emerging evidence indicates that hyperuricemia more frequently results from impaired renal uric acid excretion rather than excessive purine intake alone.^[7]^

Beyond genetic predisposition, multiple modifiable factors contribute substantially to gout development. Obesity emerges as a dominant risk factor, with individuals maintaining a body mass index (BMI) above 30 kg/m^2^ experiencing 2 to 3 times greater odds of developing gout, and approximately 31% of cases attributable to excess weight alone.^[8,9]^ Dietary patterns exert influence on gout risk: high consumption of purine-rich foods such as red meat and fructose-containing processed products increases risk, while adherence to the Dietary Approaches to Stop Hypertension diet has been associated with up to 21% risk reduction.^[9,10]^ In addition, dyslipidemia, particularly elevated triglyceride levels and reduced high-density lipoprotein (HDL) cholesterol concentrations, has been linked to hyperuricemia and gout development.^[11]^

Over the past 2 decades, the global incidence of gout has nearly doubled, paralleling the increasing prevalence of metabolic syndrome worldwide.^[12]^ However, current evidence predominantly stems from observational and cross-sectional studies, which are inherently susceptible to confounding, reverse causation, and residual bias.^[13]^ The complex interrelationships between dietary habits, lifestyle behaviors, metabolic factors, and gout risk present particular challenges for traditional epidemiological approaches, as these exposures are frequently correlated.

Given the ethical and logistical constraints of conducting randomized controlled trials for long-term dietary and lifestyle interventions, Mendelian randomization (MR) offers an alternative by using genetic variants as instrumental variables for modifiable exposures, leveraging the random allocation of alleles during meiosis to mimic a randomized trial.^[13,14]^ The validity of MR depends on 3 core assumptions: genetic variants must be robustly associated with the exposure, must not be associated with confounders, and must influence the outcome solely through the exposure.^[13,14]^ Multivariable extensions allow several correlated exposures to be examined simultaneously, which is particularly valuable where metabolic and dietary factors are intercorrelated.^[14]^

This study employed a comprehensive two-sample MR framework to investigate the causal relationships between dietary patterns, lifestyle behaviors, anthropometric measures, and lipid profiles with gout risk, using genetic data from the UK Biobank and FinnGen. A prior MR study reported a causal effect of adiposity on gout^[15]^; the present analysis extends that work by examining dietary and lifestyle exposures alongside adiposity, by testing independence through multivariable models, and by replicating findings across 2 cohorts.

## 2. Materials and methods

### 2.1. Study design

This two-sample MR study investigated causal relationships between dietary factors, lifestyle behaviors, BMI, and lipid profiles with gout risk, following established MR principles and the STROBE-MR reporting guideline.^[13,14]^

### 2.2. Study population and outcome definition

UK Biobank: Gout was defined using the Medical Research Council Integrative Epidemiology Unit genome-wide association analysis of self-reported non-cancer illness code for gout (OpenGWAS identifier ukb-b-13251), comprising 6543 cases and 456,390 controls of European ancestry (n = 462,933).^[16]^ Cases were ascertained by self-report at baseline assessment; this dataset does not incorporate linked hospital International Classification of Diseases, Tenth Revision records, and the implications are addressed in the Limitations section.

FinnGen: The analysis included 150,797 Finnish participants, comprising 3576 gout cases and 147,221 controls. Gout cases were defined using International Classification of Diseases, Tenth Revision codes M10.0 to M10.9 from national hospital discharge and death registries (OpenGWAS identifier finn-b-M13_GOUT).^[17]^ All participants provided informed consent, and the contributing studies received appropriate ethical approvals.

Effect scale: The UK Biobank association statistics were generated with a linear mixed model applied to a binary trait, so the effect estimates are on the risk-difference scale, whereas FinnGen estimates are on the log-odds scale. Exponentiated UK Biobank coefficients are therefore compressed toward the null and are not numerically comparable with FinnGen odds ratios (ORs). Comparison between cohorts is accordingly restricted to the direction and statistical significance of associations, not to effect magnitude.

### 2.3. Genetic instrumental variables

Instruments were obtained for 12 exposures from large-scale genome-wide association studies conducted by the GWAS and Sequencing Consortium of Alcohol and Nicotine Use, Genetic Investigation of Anthropometric Traits, Global Lipids Genetics Consortium, and Medical Research Council Integrative Epidemiology Unit consortia, and from Pirastu et al for dried fruit intake.^[18–22]^ The dietary instrument used here derives from a genome-wide association study (GWAS) of dried fruit consumption specifically, rather than total fruit intake, and is described as such throughout. Variants were selected at genome-wide significance (*P* < 5 × 10^−8^), except for physical activity, for which a relaxed threshold (*P* < 1 × 10^−5^) was applied because too few variants reached genome-wide significance, consistent with previous MR studies.^[14,23–25]^ All selected variants were clumped for linkage disequilibrium (*r*^2^ < 0.001, window 10,000 kb) using the 1000 Genomes European reference panel. Total cholesterol and low-density lipoprotein cholesterol instruments derive from an earlier Global Lipids Genetics Consortium release than those for HDL cholesterol and triglycerides, and sample sizes differ accordingly. Instrument characteristics are given in Table 1.

**Table 1:** Genetic instruments for the 12 exposures examined.

Exposure	GWAS accession	Sample size	SNPs (UKB/FinnGen)	Mean *F*	*R*^2^ (%)	Source
Dried fruit intake	ebi-a-GCST90096909	409,125	41/41	42	0.13	Pirastu et al
Salad/raw vegetable intake	ukb-b-1996	435,435	17/18	39	0.07	UK Biobank (MRC-IEU)
Cheese intake	ukb-b-1489	451,486	62/62	39	0.62	UK Biobank (MRC-IEU)
Smoking initiation	ieu-b-4877	632,802	84/85	42	2.23	GSCAN
Smoking intensity	ieu-b-142	250,822	22/23	101	3.43	GSCAN
Maternal smoking	ukb-b-17685	397,732	16/16	41	0.03	UK Biobank (MRC-IEU)
BMI	ieu-a-2	338,856	75/77	58	1.98	GIANT
Physical activity	ukb-b-151	440,512	11/11	40	0.37	UK Biobank (MRC-IEU)
Total cholesterol	ieu-a-782	88,311	41/44	133	6.44	GLGC
LDL cholesterol	ieu-a-781	83,032	36/38	206	9.49	GLGC
HDL cholesterol	ieu-a-299	187,077	83/85	155	7.90	GLGC
Triglycerides	ieu-a-302	176,173	54/56	160	5.08	GLGC

SNP counts are those retained after harmonization against each outcome and differ between cohorts because variants absent from one outcome dataset were dropped. *F* and *R*^2^ are shown for the UK Biobank analysis.

BMI = body mass index, GIANT = Genetic Investigation of Anthropometric Traits, GLGC = Global Lipids Genetics Consortium, GSCAN = GWAS and Sequencing Consortium of Alcohol and Nicotine Use, GWAS = genome-wide association study, HDL = high-density lipoprotein, LDL = low-density lipoprotein, MRC-IEU = Medical Research Council Integrative Epidemiology Unit, SNP = single-nucleotide polymorphism, UKB = UK Biobank.

### 2.4. Quality control and instrument strength

Single-nucleotide polymorphisms were excluded if they were palindromic with intermediate allele frequencies or could not be unambiguously aligned between exposure and outcome datasets. Exposure and outcome data were harmonized for effect allele alignment and strand orientation, and proxy single-nucleotide polymorphisms (*r*^2^ > 0.8) were substituted where target variants were unavailable. Instrument strength was quantified per variant as *F* = (β/SE)^2^. All instruments exceeded the conventional threshold of 10 (minimum *F* = 29.8), indicating that weak-instrument bias is unlikely. Variance explained was computed as the sum of 2 × effect allele frequency × (1 − effect allele frequency) × β^2^ across variants.

### 2.5. Statistical analysis

Inverse-variance weighted (IVW) regression with multiplicative random effects served as the primary analysis. This estimator was selected in preference to fixed-effects IVW because Cochran’s *Q* indicated substantial heterogeneity for most exposures, under which fixed-effects standard errors are understated. Sensitivity analyses comprised MR-Egger regression with its intercept test for directional pleiotropy, the weighted median estimator, the weighted mode estimator, and MR-Pleiotropy RESidual Sum and Outlier for outlier detection and correction. Heterogeneity was quantified by Cochran’s *Q* and *I*^2^. The applicability of MR-Egger was assessed using the *I*^2^GX statistic, since values below 0.9 indicate regression dilution that biases the MR-Egger slope toward the null.

Twenty-four tests were performed (12 exposures in 2 cohorts). Benjamini-Hochberg (BH) adjusted *P* values controlling the false discovery rate at 5% are reported alongside unadjusted values, and interpretation is based on the adjusted values. Analyses were conducted in R (version 4.2.3; R Foundation for Statistical Computing, Vienna, Austria) with the *TwoSampleMR* and *MVMR* (MRC Integrative Epidemiology Unit, University of Bristol, Bristol, UK) packages.

#### 2.5.1. Multivariable MR

To identify independent causal effects among correlated exposures, multivariable MR analyses were conducted using 3 predefined models: a metabolic model (BMI, triglycerides, HDL cholesterol); a dietary-metabolic model (dried fruit intake, cheese intake, BMI); and a lifestyle model (smoking initiation, physical activity, BMI). Instruments were variants associated with at least one exposure at *P* < 5 × 10^−8^. Conditional *F* statistics were computed for each exposure to quantify instrument strength after conditioning on the others, and *Q* statistics were used to assess instrument validity.

### 2.6. Ethical considerations

This study used only publicly available summary-level genetic data from previously published genome-wide association studies. All original studies obtained appropriate ethical approval from their respective institutional review boards and informed consent from participants. The UK Biobank received ethical approval from the Northwest Multi-Centre Research Ethics Committee (reference 11/NW/0382),^[16]^ and the FinnGen project was approved by the Finnish Institute for Health and Welfare (reference THL/1390/5.05.00/2017).^[17]^ Because only summary statistics were analyzed and no individual-level data were accessed, no additional ethical approval or informed consent was required for the present study.

### 2.7. Use of generative artificial intelligence

Generative AI was used solely for language editing. Study design, data, analysis, and interpretation were conducted entirely by the author.

## 3. Results

### 3.1. Instrument characteristics

Instruments for the 12 exposures ranged from 11 to 85 variants per exposure-outcome pair after harmonization (Table 1). All instruments were strong, with per-variant *F* statistics ranging from 29.8 to 4089.3 and mean *F* between 39.0 and 218.7. Variance explained ranged from 0.03% (maternal smoking) to 10.5% (low-density lipoprotein cholesterol). The *I*^2^GX statistic exceeded 0.9 for the lipid traits, BMI, and smoking intensity, but fell below 0.6 for dried fruit, salad, cheese, maternal smoking, and smoking initiation, and was 0.00 for physical activity, indicating that MR-Egger estimates are not informative for those exposures.

### 3.2. Univariable MR

**Figure 1.**
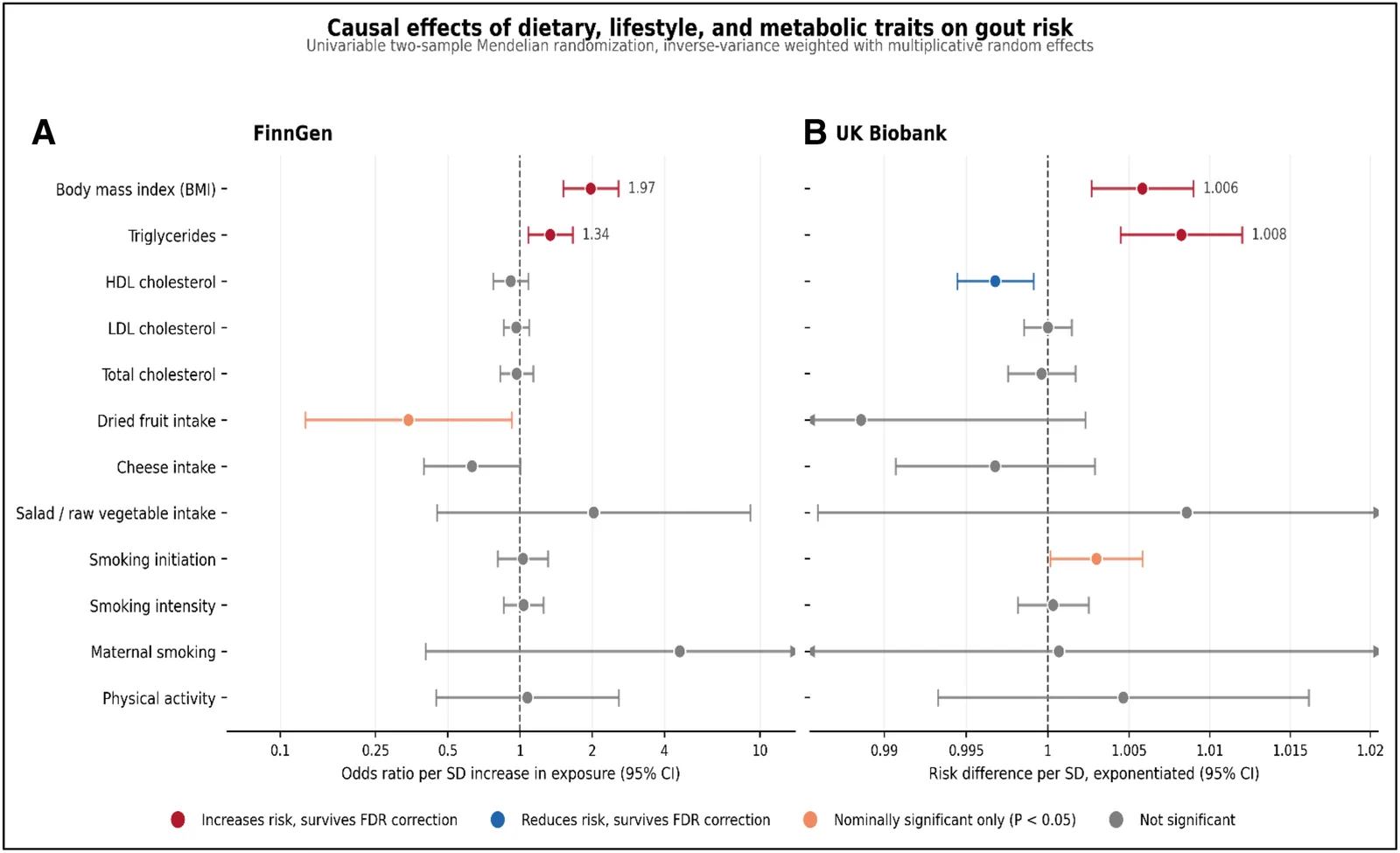
Causal effects of dietary, lifestyle, and metabolic traits on gout risk in 2 European cohorts. Estimates are from univariable two-sample Mendelian randomization using inverse-variance weighted regression with multiplicative random effects. Panel (A) shows FinnGen, in which effects are on the log-odds scale. Panel (B) shows UK Biobank, in which the outcome association statistics were generated with a linear mixed model, and effects are therefore on the risk-difference scale. The 2 panels use separate x-axes for this reason, and effect magnitudes are not comparable between them; direction and statistical significance are. Color indicates whether an association survived Benjamini-Hochberg correction across all 24 tests. Arrowheads denote CIs extending beyond the plotted range. BMI = body mass index, CI = confidence interval, FDR = false discovery rate, HDL = high-density lipoprotein, LDL = low-density lipoprotein, SD = standard deviation.

UK Biobank: BMI was associated with an increased gout risk (OR = 1.006, 95% confidence interval [CI] = 1.003–1.009, *P* < .001), as were triglycerides (OR = 1.008, 95% CI = 1.004–1.012, *P* < .001). HDL cholesterol was inversely associated (OR = 0.997, 95% CI = 0.994–0.999, *P* = .007). Smoking initiation reached nominal significance (OR = 1.003, 95% CI = 1.000–1.006, *P* = .038) but did not survive correction for multiple testing. No other exposure, including dried fruit intake (OR = 0.989, 95% CI = 0.975–1.002, *P* = .102), was associated with gout (Table 2 and Fig. 1).

**Table 2:** Univariable Mendelian randomization estimates for gout, UK Biobank (6543 cases, 456,390 controls).

Exposure	SNPs	OR (95% CI)	*P*	BH-adjusted *P*	Weighted median OR	*P*	Cochran *Q P*	Egger intercept *P*
Dried fruit intake	41	0.989 (0.975–1.002)	.102	.273	0.991	.227	<.001	.826
Salad/raw vegetable intake	17	1.009 (0.986–1.032)	.460	.736	1.025	.015	<.001	.147
Cheese intake	62	0.997 (0.991–1.003)	.299	.599	0.995	.157	<.001	.120
Smoking initiation	84	1.003 (1.000–1.006)	.038	.132	1.000	.782	<.001	.534
Smoking intensity	22	1.000 (0.998–1.003)	.770	.924	1.000	.708	.097	.591
Maternal smoking	16	1.001 (0.978–1.024)	.953	.993	1.004	.742	.078	.002
BMI	75	1.006 (1.003–1.009)	<.001	.002	1.008	<.001	<.001	.900
Physical activity	11	1.005 (0.993–1.016)	.425	.728	1.008	.043	<.001	.253
Total cholesterol	41	1.000 (0.998–1.002)	.715	.912	1.001	.238	<.001	.307
LDL cholesterol	36	1.000 (0.999–1.001)	.993	.993	1.001	.196	<.001	.878
HDL cholesterol	83	0.997 (0.994–0.999)	.007	.037	1.001	.443	<.001	<.001
Triglycerides	54	1.008 (1.004–1.012)	<.001	<.001	1.006	<.001	<.001	.540

Estimates are from inverse-variance weighted regression with multiplicative random effects. UK Biobank effect estimates are on the risk-difference scale and are compressed toward the null relative to true odds ratios; they are not numerically comparable with the FinnGen estimates in Table 3.

BH = Benjamini-Hochberg, BMI = body mass index, CI = confidence interval, HDL = high-density lipoprotein, LDL = low-density lipoprotein, OR = odds ratio, SNP = single-nucleotide polymorphism.

FinnGen: BMI showed the strongest association (OR = 1.97, 95% CI = 1.51–2.57, *P* < .001), followed by triglycerides (OR = 1.34, 95% CI = 1.08–1.66, *P* = .008). Dried fruit intake was inversely associated (OR = 0.34, 95% CI = 0.13–0.92, *P* = .034), and cheese intake approached significance (OR = 0.63, 95% CI = 0.40–1.00, *P* = .052). Neither survived correction for multiple testing. HDL cholesterol was not associated with gout in this cohort (OR = 0.91, 95% CI = 0.77–1.08, *P* = .295; Table 3 and Fig. 1).

**Table 3:** Univariable Mendelian randomization estimates for gout, FinnGen (3576 cases, 147,221 controls).

Exposure	SNPs	OR (95% CI)	*P*	BH-adjusted *P*	Weighted median OR	*P*	Cochran *Q P*	Egger intercept *P*
Dried fruit intake	41	0.34 (0.13–0.92)	.034	.132	0.34	.134	.351	.174
Salad/raw vegetable intake	18	2.03 (0.45–9.13)	.357	.658	4.79	.135	.330	.132
Cheese intake	62	0.63 (0.40–1.00)	.052	.156	1.23	.551	.376	.392
Smoking initiation	85	1.03 (0.81–1.31)	.816	.932	0.93	.698	.329	.966
Smoking intensity	23	1.04 (0.86–1.25)	.722	.912	0.98	.875	.413	.303
Maternal smoking	16	4.63 (0.40–53.15)	.218	.524	2.20	.580	.061	.921
BMI	77	1.97 (1.51–2.57)	<.001	<.001	2.08	<.001	.216	.716
Physical activity	11	1.07 (0.45–2.58)	.875	.954	1.65	.296	.012	.028
Total cholesterol	44	0.97 (0.83–1.13)	.693	.912	1.01	.911	.037	.594
LDL cholesterol	38	0.96 (0.85–1.09)	.558	.837	1.01	.945	.124	.275
HDL cholesterol	85	0.91 (0.77–1.08)	.295	.599	1.07	.514	<.001	.031
Triglycerides	56	1.34 (1.08–1.66)	.008	.037	1.12	.450	<.001	.512

Estimates are from inverse-variance weighted regression with multiplicative random effects. Benjamini-Hochberg adjusted *P* values are computed across all 24 tests (12 exposures in 2 cohorts).

BH = Benjamini-Hochberg, BMI = body mass index, CI = confidence interval, HDL = high-density lipoprotein, LDL = low-density lipoprotein, OR = odds ratio, SNP = single-nucleotide polymorphism.

Correction for multiple testing: Of the 24 tests performed, 7 were nominally significant. Three survived Bonferroni correction (BMI in both cohorts and triglycerides in UK Biobank), and 5 survived BH correction at a 5% false discovery rate, adding HDL cholesterol in UK Biobank and triglycerides in FinnGen. The dietary associations did not survive either correction.

### 3.3. Multivariable MR

BMI retained an independent association with gout in 5 of the 6 multivariable models (Table 4). In FinnGen, the BMI estimate was stable across the metabolic (OR = 1.84), dietary-metabolic (OR = 1.95), and lifestyle (OR = 2.07) models, closely matching the univariable estimate of 1.97. The same stability was seen in UK Biobank. The exception was the metabolic model in UK Biobank, in which BMI was attenuated after adjustment for triglycerides and HDL cholesterol (OR = 1.003, 95% CI = 0.999–1.008, *P* = .144), while triglycerides remained strongly associated (OR = 1.008, 95% CI = 1.005–1.011, *P* < .001).

**Table 4:** Multivariable Mendelian randomization estimates for gout across 3 predefined models.

Model	Cohort	Exposure	SNPs	OR (95% CI)	*P*	Conditional *F*
Metabolic	UK Biobank	BMI	57	1.003 (0.999–1.008)	.144	27.0
		HDL cholesterol	75	0.999 (0.997–1.002)	.517	52.5
		Triglycerides	37	1.008 (1.005–1.011)	<.001	39.8
Metabolic	FinnGen	BMI	58	1.83 (1.31–2.58)	<.001	26.9
		HDL cholesterol	77	1.01 (0.85–1.21)	.886	52.5
		Triglycerides	38	1.28 (1.02–1.60)	.032	39.4
Dietary-metabolic	UK Biobank	Dried fruit	26	0.989 (0.973–1.005)	.178	8.2
		BMI	64	1.006 (1.002–1.009)	.002	24.2
		Cheese intake	32	0.998 (0.990–1.007)	.727	7.6
Dietary-metabolic	FinnGen	Dried fruit	26	0.31 (0.09–1.09)	.068	8.1
		BMI	66	1.95 (1.46–2.61)	<.001	22.0
		Cheese intake	32	1.01 (0.50–2.03)	.986	7.5
Lifestyle	UK Biobank	BMI	61	1.008 (1.004–1.011)	<.001	27.5
		Smoking initiation	64	1.001 (0.998–1.004)	.422	21.6
		Physical activity	6	1.001 (0.995–1.008)	.651	4.2
Lifestyle	FinnGen	BMI	63	2.06 (1.56–2.73)	<.001	26.2
		Smoking initiation	63	0.98 (0.75–1.29)	.907	21.3
		Physical activity	6	1.33 (0.76–2.34)	.321	4.1

Conditional *F* statistics quantify instrument strength for each exposure after conditioning on the others in the model. Values below 10 indicate weak instruments; the physical activity estimates (conditional *F* 4.1–4.2, 6 shared variants) should be regarded as uninformative.

BMI = body mass index, CI = confidence interval, HDL = high-density lipoprotein, OR = odds ratio, SNP = single-nucleotide polymorphism.

Triglycerides retained independent significance in the metabolic model in both cohorts. Dried fruit intake was not independently associated with gout after adjustment for BMI in either cohort, and its point estimate was essentially unchanged from the univariable analysis (FinnGen: 0.34–0.31), indicating that the association is not mediated through BMI. Cheese intake, smoking initiation, and physical activity showed no independent effects.

Conditional *F* statistics exceeded 20 for BMI, smoking initiation, HDL cholesterol, and triglycerides in all models. They fell below the conventional threshold of 10 for dried fruit (8.1–8.2) and cheese intake (7.5–7.6) and were lowest for physical activity (4.1–4.2, based on only 6 shared variants), so the multivariable estimate for physical activity should be regarded as uninformative rather than null.

### 3.4. Sensitivity analyses

Agreement across estimators was strongest for BMI, which was directionally consistent and nominally significant under IVW, weighted median, and weighted mode in both cohorts. For triglycerides in UK Biobank, all 4 estimators agreed. For the remaining exposures, including dried fruit and cheese, the median- and mode-based estimators did not reproduce the IVW result, so those associations rest on the IVW estimate alone.

The MR-Egger intercept differed significantly from zero for HDL cholesterol in both cohorts (*P* = .031 and *P* < .001), for physical activity in FinnGen (*P* = .028), and for maternal smoking in UK Biobank (*P* = .003), indicating directional pleiotropy for those exposures. Cochran *Q* indicated significant heterogeneity for most exposures, particularly in UK Biobank, which motivated the use of multiplicative random effects as the primary model. MR-Pleiotropy RESidual Sum and Outlier identified outlying variants in 12 of the 24 analyses, with up to 6 outliers for triglycerides in UK Biobank.

Leave-one-out analysis confirmed that no single variant drove the BMI or triglyceride associations in either cohort. For dried fruit in FinnGen, the estimate ranged from 0.29 to 0.45 across iterations, and the CI included the null in 7 of 41 iterations, indicating a less stable association.

## 4. Discussion

This two-sample MR analysis provides genetic evidence that BMI is the dominant modifiable causal determinant of gout, with triglycerides contributing independently. Both associations replicated across 2 large European cohorts and survived correction for multiple testing. Dietary associations were weaker, cohort-specific, and did not withstand correction, and are therefore presented as hypothesis-generating rather than established.

### 4.1. BMI as the dominant causal factor

BMI was the most consistent causal determinant of gout in this analysis. Its effect was directionally concordant in both cohorts, survived the strictest correction for multiple testing, and was reproduced by the weighted median and weighted mode estimators. Most importantly, the BMI estimate was stable under multivariable adjustment: in FinnGen it moved only between 1.84 and 2.07 across 3 models containing different co-exposures, against a univariable estimate of 1.97. This stability is the strongest available evidence that BMI acts on gout risk directly rather than as a proxy for correlated metabolic or dietary traits.

These findings corroborate observational evidence that obese individuals face substantially higher gout risk.^[26,27]^ Mechanistically, increased adiposity contributes to hyperuricemia through several interconnected pathways: insulin resistance reduces renal uric acid clearance, chronic low-grade inflammation enhances purine catabolism, and visceral adipose tissue produces inflammatory mediators that impair renal function.^[28]^ Genetic studies have identified shared loci, including GCKR and PDZK1, that associate with both adiposity and serum urate.^[29]^ The magnitude of the effect is consistent with a previous MR study of adiposity and gout.^[15]^

One informative exception emerged. In UK Biobank, BMI was attenuated to nonsignificance once triglycerides and HDL cholesterol were included in the same model, while triglycerides remained strongly associated. In FinnGen, the opposite pattern held, with BMI dominant. This suggests that the metabolic cluster and adiposity share a substantial causal pathway, and that their relative contributions may differ between populations. It also argues against treating any single metabolic parameter as the sole intervention target.

### 4.2. Triglycerides and metabolic pathways

Elevated triglycerides showed a causal association with gout in both cohorts and retained independent significance in the multivariable metabolic model in both. These findings are consistent with observational studies reporting higher hyperuricemia risk at higher triglyceride concentrations.^[30]^ Mechanistically, hypertriglyceridemia disrupts free fatty acid metabolism and accelerates adenosine triphosphate degradation, promoting uric acid synthesis through enhanced xanthine oxidase activity.^[31]^ The clinical relevance of this pathway is supported by evidence that lipid-lowering therapy can reduce serum urate, although direct effects on gout incidence require further investigation.^[32]^

### 4.3. HDL cholesterol

HDL cholesterol was inversely associated with gout in UK Biobank and survived correction for multiple testing but was not associated in FinnGen and did not retain independent significance in the multivariable metabolic model in either cohort. This result requires particular caution: the MR-Egger intercept indicated directional pleiotropy for HDL cholesterol in both cohorts, and the weighted median estimator did not reproduce the association. HDL cholesterol is strongly genetically correlated with triglycerides, and the multivariable results suggest that the univariable HDL signal may partly reflect that correlation. The proposed mechanisms, involving anti-inflammatory activity and effects on renal urate transport,^[33,34]^ remain plausible but are not established by the present data.

### 4.4. Dietary exposures

Dried fruit intake was inversely associated with gout in FinnGen but not in UK Biobank, and the association did not survive correction for multiple testing. It was also not reproduced by the weighted median or weighted mode estimators, and leave-one-out analysis showed the CI crossing the null when certain variants were removed. The finding is therefore suggestive rather than established.

If real, the mechanism is not obvious. Dried fruit is a concentrated source of fructose, which raises serum urate,^[35]^ so a protective effect runs counter to the naive prediction. Ascorbic acid, which enhances renal urate excretion,^[36]^ is largely degraded during drying, making it an unlikely explanation. Polyphenolic compounds, which are retained or concentrated by drying, are a more plausible candidate, as is confounding by the broader dietary pattern of which dried fruit consumption is a marker. Notably, the association was not attenuated by adjustment for BMI in the multivariable model, indicating that it is not mediated through adiposity.

Cheese intake approached but did not reach significance in FinnGen (*P* = .052) and showed no association in UK Biobank. The weighted median estimate was directionally opposite to the IVW estimate, and the conditional *F* statistic in the multivariable model was below 10. No claim of a protective dairy effect is supported by these data. Salad intake showed no association in either cohort.

### 4.5. Lifestyle exposures

Smoking initiation reached nominal significance in UK Biobank but did not survive correction for multiple testing, was not reproduced by any other estimator, and lost significance in the multivariable lifestyle model. Smoking intensity and maternal smoking showed no association in either cohort. Physical activity showed no association, but its instrument was weak in the multivariable setting (conditional *F* = 4.1–4.2 on 6 variants), so the absence of an effect should not be interpreted as evidence of no effect.

### 4.6. Strengths and limitations

The principal strengths of this study are the use of genetic instruments to reduce confounding and exclude reverse causation, replication across 2 large independent cohorts, the application of multivariable models to distinguish independent from mediated effects, and a comprehensive set of sensitivity analyses.

Several limitations warrant emphasis. First, all analyses were restricted to European ancestry populations. This reduces population stratification bias but limits generalizability to other ancestral groups, in whom both gout prevalence and dietary patterns differ substantially. Trans-ancestry MR is a priority for future work.

Second, the dietary and lifestyle exposures derive from self-reported measures in the original GWAS, and the UK Biobank gout outcome is based on self-report at baseline rather than linked hospital records. Both introduce measurement error. Where such misclassification is non-differential with respect to genotype, which is expected here because genotype is fixed at conception and cannot be influenced by reporting behavior, the resulting bias is toward the null, so the estimates reported here are likely conservative rather than inflated. Nonetheless, self-reported outcome ascertainment may underestimate case numbers and reduce statistical power.

Third, 24 statistical tests were performed. BH adjusted *P* values are reported throughout, and interpretation is based on them, but the nominally significant associations that did not survive correction, particularly the dietary findings, should be treated as hypothesis-generating and require independent replication.

Fourth, several exposure GWAS were conducted in UK Biobank, and the dried fruit GWAS also drew on UK Biobank participants. These overlap with the UK Biobank outcome dataset, which biases two-sample estimates toward the confounded observational association. The bias is limited by the strength of the instruments used but is not eliminated. The FinnGen analyses are free of sample overlap, since no exposure GWAS included Finnish participants, and the concordance of the BMI and triglyceride findings across the 2 cohorts argues against overlap alone explaining them.

Fifth, the analysis examined individual food items rather than dietary patterns. Single items may not capture the synergistic and cumulative effects of real-world eating behavior, and the dried fruit instrument in particular may partly index a broader dietary pattern. GWAS of composite dietary indices would allow this to be addressed directly.

Finally, the 2 cohorts report effects on different scales, so only the direction and significance of associations can be compared between them, not their magnitudes. MR-Egger was uninformative for several exposures because of regression dilution, as quantified by the *I*^2^GX statistic.

## 5. Conclusions

This MR analysis provides genetic evidence that BMI is the dominant modifiable causal determinant of gout, with triglycerides contributing independently. Both findings replicated across 2 large European cohorts, were stable under multivariable adjustment, and survived correction for multiple testing. Dietary associations, including the inverse association of dried fruit intake in FinnGen, were weaker and did not withstand correction, and should be regarded as hypothesis-generating.

These results support prioritizing weight management and metabolic health in gout prevention, rather than single-nutrient dietary advice, and identify adiposity as the intervention target with the strongest causal support. Replication in non-European populations and in cohorts with objectively ascertained gout outcomes remains necessary.

## Author contributions

**Conceptualization:** Mohammad A. Jareebi.

**Data curation:** Mohammad A. Jareebi.

**Formal analysis:** Mohammad A. Jareebi.

**Investigation:** Mohammad A. Jareebi.

**Methodology:** Mohammad A. Jareebi.

**Project administration:** Mohammad A. Jareebi.

**Resources:** Mohammad A. Jareebi.

**Software:** Mohammad A. Jareebi.

**Supervision:** Mohammad A. Jareebi.

**Validation:** Mohammad A. Jareebi.

**Visualization:** Mohammad A. Jareebi.

**Writing – original draft:** Mohammad A. Jareebi.

**Writing – review & editing:** Mohammad A. Jareebi.
